# Efficacy and safety of sugammadex in patients undergoing renal transplantation

**DOI:** 10.1186/s40981-018-0192-z

**Published:** 2018-07-25

**Authors:** Yasumasa Ono, Yoshihito Fujita, Takahiro Kajiura, Hazuki Okawa, Juntaro Nakashima, Hideo Isobe, Yoshihiro Fujiwara

**Affiliations:** Department of Anesthesiology, Aichi Medical University School of Medicine, 1-1 Yazako Karimata, Nagakute, Aichi 480-1195 Japan

**Keywords:** Rocuronium, Sugammadex, Renal transplantation, Safety, Efficacy

## Abstract

**Background:**

Sugammadex reverses rocuronium by encapsulating it, creating a stable complex that is mainly excreted by the kidneys. Nonetheless, in view of exposure to sugammadex during renal transplantation, current safety data are insufficient. We retrospectively investigated the safety and efficacy of sugammadex in the immediate perioperative period and over long-term follow-up.

**Case presentation:**

We studied 99 consecutive patients who underwent living renal transplantation. We investigated the efficacy of sugammadex and its perioperative complications in the first 48–72 h in the surgical intensive care unit and in the follow-up for 6 months.

Before transplantation, 53 patients required hemodialysis. The median serum creatinine concentration was 5.6 mg/dl, and blood urea nitrogen (BUN) was 30 mg/dl. During surgery, the median rocuronium and sugammadex dose was 160 mg (interquartile range 130–185 mg) and 200 mg (200–200 mg), respectively. After transplantation, the median serum creatinine concentration was 2.4 mg/dl at postoperative day 1, and BUN was 21 mg/dl, respectively. No adverse events were recorded during the observation period.

**Conclusion:**

We investigated whether 99 consecutive patients undergoing renal transplantation may benefit from the use of sugammadex. There were no adverse events. We concluded that, in our observational period, sugammadex was efficacious and safe in patients who underwent renal transplantation.

## Background

Sugammadex reverses rocuronium by encapsulating it, creating a stable complex that is mainly excreted by the kidneys. Although sugammadex clearance is reduced in renal impairment [1], case reports have described the safe use of rocuronium and sugammadex in renal transplantation [2]. Nonetheless, in view of exposure to sugammadex during renal transplantation, current safety data are insufficient. We retrospectively investigated the safety and efficacy of sugammadex in the immediate perioperative period and over long-term follow-up.

## Case presentation

We studied 99 consecutive patients who were diagnosed with severe renal failure and underwent living renal transplantation under general anesthesia at our institution between March 2011 and June 2016 (Tables 1 and 2). Ethical approval for this study (2016-H281) was provided by the Ethical Committee of Aichi Medical University, Nagakute, Japan (Chairperson Prof M. Hanyuuda) on 19 December 2016. Obtaining written informed consent from each individual was waived from the Ethical Committee because of its retrospective manner and publication of its information on the website of the hospital. We investigated the efficacy of sugammadex and recorded perioperative complications in the first 48–72 h in the surgical intensive care unit. We followed up all patients for longer than 6 months.

**Table 1:** Patient characteristics and renal function

Age (years)	53 (43–61)
Height (m)	166 (158–170)
Weight (kg)	59 (51–69.5)
BMI (kg/m^2^)	22 (20–24)
Sex (F/M)	63 (63.6%), 36 (36.4%)
Creatinine (mg/dl)	5.6 (4.5–7.5)
BUN (mg/dl)	30 (24.5–34.5)
eGFR (ml/min/1.73mm^3^)	8 (6–11)
Dialysis episode (number)	
None or < 1 month	53 (53.5%)
< 12 months	19 (19.2%)
≧ 12 months	27 (27.3%)
Anesthesia (min)	425 (382–459.5)
Surgery (min)	317 (290–358)
Rocuronium dose (mg)	160 (130–185)
Sugammadex dose (mg)	200 (200–200)

Values are median (interquartile range) or number (%)

*BMI* body mass index, *BUN* blood urea nitrogen, *eGFR* estimated glomerular filtration rate

**Table 2:** Renal function and complication after surgery

	Day 1	Day 3	Day 5	Discharge	≧ 6 months
Renal function after surgery					
Creatinine	2.4(1.75, 3.45)	1.3(0.9, 1.6)	1.2(1.0, 1.5)	1.2(1.0, 1.6)	1.4(1.1, 1.6)
BUN	21(17, 27)	18(14, 24)	20(16, 28)	17(13.5, 21)	21(18, 25)
eGFR	22(14.5, 30.5)	48(35.5, 58)	47(38.5, 57.5)	45(38, 55.5)	40(33, 49)
Complication with muscle relaxants					
72 h in ICU	0				
Following period	0				

Values are median (interquartile range)

*BUN* blood urea nitrogen, *eGFR* estimated glomerular filtration rate

In all patients, anesthesia was maintained with either sevoflurane or desflurane and intravenous opioids. Intraoperative anesthetic management was performed by the attending anesthesiologist who had more than 5 years of experience. Generally, propofol (1–1.5 mg/kg) and rocuronium (0.6–0.9 mg/kg) were used for tracheal intubation. Adjustment of rocuronium was determined by the attending anesthesiologist with monitoring by acceleromyography (TOF-Watch SX; Organon Ireland Ltd., a division of MSD, Dublin, Ireland). Train-of-four (TOF) ratio was kept to count 0 or 1. Standard monitoring was performed, and core rectal temperature was maintained above 36.0 °C throughout the surgery with a forced-air warmer blanket. For immunosuppression, 20 mg basiliximab at the start of surgery and 500 mg methylprednisolone before anastomosis of the renal artery and vein were administered. After surgery, the attending anesthesiologist determined the adequacy of neuromuscular transmission by clinical signs, a bucking reaction against the endotracheal tube, spontaneous breathing, and movement of extremities in response to commands. A total of 2–4 mg/kg sugammadex was administered, and the tracheal tube was extubated with confirmed clinical signs and a TOF ratio > 0.9. The patients were transferred to the surgical intensive care unit (SICU) and had preoperative care until day 2 or 3. In the SICU, standard monitoring and a daily blood test were performed, and oral immunosuppression drugs were administered.

The primary safety variable was the occurrence of postoperative complications related to recurarization, such as upper airway obstruction requiring mechanical intervention, hypoxemia (SpO2 < 90) with 10 l/min supplemental oxygen administration with mask, need for tracheal reintubation, and muscular weakness until hospital discharge, and more than a 6-month follow-up period. We investigated these signs and symptoms described above from medical records during the stay in the SICU and in-hospital. We interviewed the attendant doctor for ambulatory care and checked medical records after hospital discharge. The secondary variable was renal function after surgery.

The median age, height, and weight of the cohort were 53 years, 1.66 m, and 59.0 kg, respectively. There were 63 (36.4%) men. Fifty-three patients required dialysis for < 1 month (53.5%), 19 for < 1 year (19.2%), and 27 for > 1 year (27.3%). The median serum creatinine concentration before transplantation was 5.6 mg/dl, blood urea nitrogen (BUN) was 30 mg/dl, and the estimated glomerular filtration rate (eGFR) was 8 ml/min/1.73mm^2^. The median duration of anesthesia and surgery was 425 and 317 min, respectively. The median rocuronium dose was 160 mg (interquartile range 130–185 mg). Three (3.0%) patients were administered sugammadex < 200 mg, one (1%) was administered 250 mg, one (1%) was administered 280 mg, two (2.0%) were administered 300 mg, two (2.0%) were administered 400 mg, and 89 (89.9%) were administered 200 mg. One patient was not administered sugammadex because the patient was not extubated at the operation room owing to deterioration of oxygenation. This patient had moderate renal insufficiency and congestive heart failure. At postoperative day 2, he was successfully extubated without sugammadex.

The median serum creatinine concentration after transplantation was 2.4 mg/dl at postoperative day 1 and 1.4 mg/dl at postoperative day 3, BUN was 21 and 18 mg/dl, and eGFR was 47 and 48 ml/min/1.73mm^2^, respectively. No adverse events were recorded during the observation period.

## Discussion

We investigated whether 99 consecutive patients undergoing renal transplantation may benefit from the use of sugammadex over a long period. Efficacy of rocuronium and sugammadex, that is muscle reluctant and its reversal, was obtained in our patients. There were no adverse events. We concluded that, in our observational period, sugammadex was efficacious and safe in patients who underwent renal transplantation.

In patients with end-stage renal failure, 4 mg/kg of sugammadex reverses deep neuromuscular blockade (NMB) [1, 3]. In our observational period, two patients needed another dose of sugammadex (total dose 5.17 and 5.19 mg/kg) after the first 200-mg sugammadex administration for complete recovery. These patients did not obtain complete recovery and their first sugammadex administrations did not reach 4 mg/kg. No patients required additional sugammadex administration at a total amount of more than 4 mg/kg. Therefore, our data agree with the findings of 4 mg/kg sugammadex for obtaining recovery from deep NMB with renal impairment [1, 3].

In our study, no adverse events were recorded after obtaining complete recovery. Fourteen (14.3%) patients had severe renal impairment (eGFR < 30) at postoperative day 5. However, there were no signs of recurarization. A sugammadex–rocuronium complex might exist in equilibrium in a low dissociation constant because of strong binding [4]. Additionally, in severe renal failure, the clearance of sugammadex is reduced by 17-fold and the elimination half-time is increased by 15-fold [5, 6]. If recurarization had happened, we thought that the sugammadex–rocuronium complex might detach for a longer period due to limited excretion. In our setting after renal transplantation, the sugammadex–rocuronium complex might excrete without detachment. In renal transplant patients, the sugammadex–rocuronium complex might remain stable for a long time.

In most of our cases the patients were administered 200 mg of sugammadex at first time without titrating recovery and patients almost obtained enough recovery. Because more amounts of sugammadex could obtain stronger recovery, the many attending anesthesiologists might use full amount of one vial of sugammadex, 200 mg. However, the main complication of sugammadex is the anaphylactic reaction and is estimated at 29 per 1,000,000 of the population [7]. In our setting, no symptoms associated with allergic reaction were observed. We should pay attention to the titrating amount of sugammadex.

The present findings have limitations. We consider that our study of 98 successfully treated cases had enough power to show the long-term efficacy and safety of rocuronium and sugammadex in patients undergoing renal transplantation because of no complications (0%, 95% confidence interval 0–3.0%). However, our study was not a controlled, pharmacokinetic, and pharmacodynamic study [8].

### Conclusion

We investigated whether 99 consecutive patients undergoing renal transplantation may benefit from the use of sugammadex. There were no adverse events. In our observational period, sugammadex appears to be safe and efficacious in patients undergoing renal transplantation.

## Abbreviations

BUN: Blood urea nitrogen
eGFR: Estimated glomerular filtration rate
NMB: Neuromuscular blockade
SICU: Surgical intensive care unit
TOF: Train-of-four
